# Neurophysiological Evidence of Compensatory Brain Mechanisms in Early-Stage Multiple Sclerosis

**DOI:** 10.1371/journal.pone.0136786

**Published:** 2015-08-31

**Authors:** Mariana López-Góngora, Antonio Escartín, Saul Martínez-Horta, Ramón Fernández-Bobadilla, Luis Querol, Sergio Romero, Miquel Àngel Mañanas, Jordi Riba

**Affiliations:** 1 Multiple Sclerosis Unit, Neurology Department, Hospital de la Santa Creu i Sant Pau, Universitat Autònoma de Barcelona, Barcelona, Spain; 2 Departament de Medicina, Universitat Autónoma de Barcelona, Barcelona, Spain; 3 Multiple Sclerosis Research Group, Biomedical Research Institute (IIB-Sant Pau), Barcelona, Spain; 4 Movement Disorders Unit, Neurology Department, Hospital de la Santa Creu i Sant Pau, Universitat Autònoma de Barcelona, Barcelona, Spain; 5 Parkinson’s Disease and Movement Disorders Research Group, Biomedical Research Institute (IIB-Sant Pau), Barcelona, Spain; 6 Department of Automatic Control (ESAII), Biomedical Engineering Research Center (CREB), Universitat Politècnica de Catalunya, BarcelonaTech (UPC), Barcelona, Spain; 7 CIBER de Bioingeniería, Biomateriales y Nanomedicina (CIBER-BBN), Barcelona, Spain; 8 Human Neuropsychopharmacology Group, Biomedical Research Institute (IIB-Sant Pau), Barcelona, Spain; 9 Centre d’Investigació de Medicaments, Servei de Farmacologia Clínica, Hospital de la Santa Creu i Sant Pau, Universitat Autònoma de Barcelona, Barcelona, Spain; 10 Departament de Farmacologia i Terapèutica, Universitat Autònoma de Barcelona, Barcelona, Spain; 11 Centro de Investigación Biomédica en Red de Salud Mental, CIBERSAM, Barcelona, Spain; Institute Biomedical Research August Pi Sunyer (IDIBAPS)—Hospital Clinic of Barcelona, SPAIN

## Abstract

Multiple sclerosis (MS) is a chronic central nervous system disorder characterized by white matter inflammation, demyelination and neurodegeneration. Although cognitive dysfunction is a common manifestation, it may go unnoticed in recently-diagnosed patients. Prior studies suggest MS patients develop compensatory mechanisms potentially involving enhanced performance monitoring. Here we assessed the performance monitoring system in early-stage MS patients using the error-related negativity (ERN), an event-related brain potential (ERP) observed following behavioral errors. Twenty-seven early-stage MS patients and 31 controls were neuropsychologically assessed. Electroencephalography recordings were obtained while participants performed: a) a stop task and b) an auditory oddball task. Behavior and ERP measures were assessed. No differences in performance were found between groups in most neuropsychological tests or in behavior or ERP components in the auditory oddball task. However, the amplitude of the ERN associated with stop errors in the stop task was significantly higher in patients. ERN amplitude correlated positively with scores on the Expanded Disability Status Scale and the Multiple Sclerosis Severity Score, and negatively with the time since last relapse. Patients showed higher neuronal recruitment in tasks involving performance monitoring. Results suggest the development of compensatory brain mechanisms in early-stage MS and reflect the sensitivity of the ERN to detect these changes.

## Introduction

Multiple sclerosis (MS) is a chronic, central nervous system (CNS) disorder characterized by white matter inflammation, demyelination and neurodegeneration [1]. Among the various clinical manifestations of MS, cognitive dysfunction is common, affecting around 50% of patients [2]. It significantly disrupts instrumental everyday activities, work, social interaction, and overall quality of life [3,4]. Deficits most commonly observed in cognitive function are related to memory, verbal fluency, speed of information processing, and visuo-spatial and executive functions [5].

Studies with recently diagnosed MS patients, including subjects with clinically isolated syndrome (CIS), have detected cognitive impairment at the onset of the disease. Potagas et al found that 27% of patients with CIS and 40% of patients with relapsing remitting multiple sclerosis (RRMS) had some degree of cognitive impairment [6], mainly in complex attention and processing speed. Incipient cognitive impairment has even been observed as early as one month after the first neurological symptom. Achiron and Barak found that 19.4% of patients assessed shortly after the first neurological symptom failed one neuropsychological test and 34.3% failed two tests. Nevertheless, this impairment had no effect on daily living activities [7].

Several authors have postulated that the development of compensatory mechanisms could help ameliorate cognitive deficits in the initial stages of the disease [8–10]. While the exact nature of these mechanisms remains largely unknown, fMRI studies point to the recruitment of additional brain areas. One study involving an attentional task and three participant groups—healthy controls, mildly cognitively impaired patients, and severely cognitively impaired patients- showed extended brain activation areas in the mildly impaired patient sample. This population showed behavioral performance that was analogous to that of the control group, but BOLD signals were larger and more widespread. The increased BOLD responses were observed over frontal and parietal brain areas and were absent in patients with severe cognitive impairment [9]. Increased activation in the early-stage patients was also observed within the frontal lobes in the dorsolateral prefrontal and anterior cingulate, both of which play a relevant role in performance monitoring [11].

Evidence of frontally-mediated compensatory mechanisms is also provided by neurophysiological studies. In an investigation using the P300 event-related brain potential (ERP) and a sample of 89 MS patients and their controls, the patient group showed an enhanced P300 frontal amplitude in a choice reaction time task [10]. Interestingly, the amplitude of the P300 correlated positively with cognitive performance in the patient group but not in the controls.

In the last two decades, the performance monitoring system has been studied intensively using more specific neurophysiological measures than the P300, such as the error-related negativity or ERN. This component of the ERP is observed following behavioral errors [12]. The ERN has been interpreted as a correlate of the error detection process [13]. It has a frontocentral topography and its generators have been located in the anterior cingulate cortex and adjacent structures in the frontal lobe [14].

In the present study we postulated the implementation of compensatory neural mechanisms in early-stage MS. To test this hypothesis we assessed the functionality of the performance monitoring system in these patients using two neurophysiological measures. We recorded the electroencephalogram and we measured the P300 (P3b and P3a) and the ERN while participants performed two different behavioral tasks. We postulated that the greater specificity of the ERN as a performance monitoring correlate would allow us to detect compensatory mechanisms in the early stages of the disease even in the absence of modifications in the P300.

## Materials and Methods

### Participants

Patients regularly attending the Multiple Sclerosis Unit at Hospital de la Santa Creu i Sant Pau were prospectively recruited from May 2011 to May 2013. Inclusion criteria were: relapsing-remitting MS (RRMS) patients diagnosed according to modified McDonald’s criteria [15] and less than 3 years of evolution since diagnosis. Participants were excluded if they presented any other neurological or psychiatric condition, and if they had a previous history of head trauma or drug abuse including alcohol. We also excluded patients with motor or sensory defects that could interfere with the administration of the task and those who had a relapse or had taken corticosteroid treatment in the previous month. A group of healthy individuals, carefully matched by age, education and sex, were also included into the study.

### Protocol approval and patient consent

All the procedures conducted were previously approved by the Ethics Review Board of Hospital de la Santa Creu i Sant Pau and each participant signed an informed consent.

### Neurological assessment

Medical history and current clinical status were assessed in all participants by the same neurologist specialized in MS (AE).Clinical status was defined by the Expanded Disability Status Scale (EDSS) [16]. Additionally, the Multiple Sclerosis Severity Score (MSSS) was obtained [17].

### Neuropsychological assessment

Cognition and behavior were assessed by a neuropsychologist specialized in MS (ML-G). Neuropsychological assessment was performed using the Spanish version of the Brief Repeatable Battery of Neuropsychological Tests [18] (BRB-N). This battery includes the following tests:

1. Selective Reminding Test (SRT) that assesses verbal memory acquisition and delayed recall. Three measurements are obtained: 1) long-term storage 2) long term retrieval and 3) the number of words recalled after the delay interval.
2. The 10/36 Spatial Recall Test which measures visual memory acquisition and delayed recall. The score is the total number of correct answers after the three trials and after the delay interval.
3. Symbol Digit Modalities Test (SDMT) that assesses attention and complex visual scanning. The score is the number of correct substitutions. The oral form was used.
4. Paced Auditory Serial Addition Test (PASAT) that measures attention, information processing speed and working memory. The score is the total number of correct answers.
5. Word List Generation (WLG) that assesses semantic verbal fluency. The subject has to produce as many words as possible of a given category (fruits and vegetables) in 90 seconds. The score is the total number of correct answers.
6. Finally, beside the WLG from the BRB-N, we administered the phonetic verbal fluency test. The score is the total number of correct answers.

### Assessment of depression and fatigue

As cognitive performance may be influenced by depression and fatigue [4] all participants also answered specific questionnaires for these symptoms. To assess depression we used the Beck Depression Inventory or BDI [19], a 21-item self-report instrument designed to measure the presence of symptoms of depression. To assess fatigue we used the Fatigue Severity Scale (FSS) [20], a 9-item scale that rates severity and the impact of fatigue on the patient’s life.

### Event-Related Brain Potentials

#### Stop task

We used a modified version of the Eriksen flanker task [21]. Participants were required to focus on the arrow in the center of an array of five arrows, designated “target”, and to respond signaling with the right hand after a right-directed arrow and with the left hand after a left-directed arrow. The four surrounding arrows either favored the target response (compatible trials, →→→→→ or←←←←←) or primed the other response (incompatible trials, →→←→→ or ←←→←←). The task included 33% of compatible trials and 50% of incompatible trials. In the remaining 17% of trials we included “stop” trials. In these trials the central green arrow changed to red (for instance: ←←←←←) after a delay of 150ms and participants had to inhibit the response in these trials. Duration of the stimuli was 300 ms. A random SOA between 900 ms and 1100 ms was used. The experiment proper consisted of 3 blocks of 4 minutes and 200 stimuli each. A 30-second rest period was allowed between blocks. Subjects were required to respond to the stimuli as fast as possible and inhibit their responses whenever a stop trial appeared.

#### Auditory oddball task

We used a modified auditory oddball task including unexpected novel, as described by Marco-Pallarés and coworkers [22]. Subjects were instructed to respond to infrequent target tones with the following characteristics: 1620 Hz, 60-msduration, 5-ms rise/fall times and an intensity of 60 dB sound pressure level (SPL). These stimuli occurred with a probability of 0.1and were embedded in a stream of standard tones of lower pitch: 1500 Hz, 60-ms duration, 5-ms rise/fall times and 60 dB SPL. These standard tones occurred with a probability of 0.8. In addition, irrelevant natural novel sounds, such as the barking of a dog or the honking of a car, were delivered with a probability of 0.1. The duration of novel sounds was between 120 and 410 ms and had an intensity of 60 dB SPL. Subjects were instructed to ignore standard and novel tones and to respond as quickly and accurately as possible with their right index finger to target tones. The duration of the task was 15 minutes.

### Electrophysiological Recording and processing

The electroencephalogram (EEG) was recorded from 19 standard scalp sites (Fp1/2, F3/4, C3/4, T3/4, T5/6, P3/4, O1/2, F7/8, Fz, Cz, Pz) using passive tin electrodes mounted in an elastic cap and referenced to the two mastoid leads. Vertical eye movements were monitored using a bipolar montage with two electrodes linked together and placed below each eye referenced to a third electrode placed centrally above the eyes. Horizontal eye movements were monitored using two electrodes placed on the external canthi of each eye. Electrode impedances were kept below 5 kOhm. The electrophysiological signals were filtered with a bandpass of 0.1–35 Hz and digitized at a rate of 250 Hz.

To maximize the information available for the subsequent event-related potential analysis (ERPs), raw EEG signals were subjected to an ocular artifact minimization process based on Blind Source Separation (BSS). This technique expresses a set of signals as a linear combination of statistically independent component signals. For this purpose, the SOBI algorithm [23] was used. This algorithm is based on an eigenvalue decomposition of time-delayed covariance matrices. After identifying the source signals associated with eye movements, corrected EEG signals were obtained from the remaining components. Identification of ocular signal sources was based on frequency and scalp topography analyses as previously described [24].The algorithm was implemented using Matlab.

Following the artifact minimization procedure, signals were processed using Brain Vision Analyzer software. Stimulus-locked ERPs were obtained for the auditory oddball task. The continuous EEG recording was segmented in epochs of 1024 ms starting 100 ms prior to stimulus presentation until 924 ms post-stimulus. Epochs were baseline-corrected, subtracting the mean amplitude in the 100 ms before stimulus presentation. A two-step artifact rejection procedure was then used. First epochs were rejected if the signal in any of the 19 channels showed amplitude values greater than ±300 μV. Subsequently, additional epochs were excluded if amplitude values were greater than ±75 μV in any of the following channels: Fz, Cz and Pz. After these preprocessing steps, three types of trials were averaged separately: epochs containing frequent stimuli, epochs containing infrequent stimuli, and epochs containing novel stimuli. These averages were obtained for each study participant and the ERP components were identified and quantified. The P3b was identified as the most positive deflection in the ERP between 300 and 600 ms post-stimulus in the infrequent trials. The P3a was identified as the most positive deflection in the ERP between 240 and 500 ms post-stimulus in the novel trials. The peak value relative to pre-stimulus baseline was calculated at Cz and Pz for the P3b (centro-parietal distribution) and at Fz and Cz for the P3a. Latencies were defined as the time taken to reach the peak value after stimulus presentation. Peak and latency values were introduced into the statistical analysis (see section 2.5.4.).

Response-locked ERPs were obtained for the stop task. The continuous EEG was segmented in epochs of 1024 ms, starting 200 ms prior to: a) commission of stop errors, and b) correct responses, until 824 ms thereafter. Thus, the 0 time point corresponded to: a) a button press when the participant should have withheld the response, and b) the emission of a correct response. Baseline correction was performed subtracting the mean amplitude in the 50 ms before button press. Subsequently, the same two-step artifact rejection and procedure described above was used. Following preprocessing, the epochs were averaged in order to obtain the ERN wave following commission errors and the absence thereof following correct responses.

Again, these averages were obtained for each study participant and the ERN was identified and quantified. The ERN was identified as the negative deflection in the ERP appearing between 0 and 100 ms following a commission error. Two quantification methods were used at Fz and Cz, because the ERN shows a frontocentral distribution. First, the average voltage was calculated between 0 and 100 ms following commission errors and correct responses. Second, peak values for the ERN were obtained relative to the pre-response baseline. This second method was only used for stop errors, as correct responses show no ERN. The obtained average and peak values were introduced into the statistical analysis described below.

A series of behavioral variables were obtained in the auditory oddball and stop tasks:

Auditory oddball task: a) percentage of non-responded target stimuli; b) percentage of erroneously responded novel stimuli.

Stop task: a) the total number of emitted responses; b) the percentage of trials responded to correctly; c) reaction times of correct responses; d) reaction times of error correction; e) the percentage of stop signal commission errors; f) post stop-error slowing; g) the difference in reaction time between incompatible and compatible trials; h) the difference in the percentage of errors between incompatible and compatible trials.

### Statistical analysis

Data are presented in summaries as means±standard deviation (SD). Demographic, clinical, neuropsychological and behavioral data were analyzed using independent samples, Student’s t tests and the χ^2^ test (gender distribution). ERP data from the auditory oddball task were analyzed using the within-subjects Condition factor (standard/target/novel) and the between-subjects Group factor (MS vs. controls). Analysis of ERP data from the stop task were analyzed using the within-subjects Condition factor (correct/error) and the between-subjects Group factor (MS vs. controls). Pairwise post-hoc comparisons were conducted using independent samples t tests. Results were considered significant for p values <0.05

## Results

### Demographic and clinical data

The study included 27 patients with diagnosis of RRMS and 31 healthy controls matched for age, sex and years of education (see Table 1). Twenty patients were receiving immunomodulators, and 7 had not yet initiated treatment. Of those receiving immunomodulators, 1 was additionally taking carbamazepine to treat trigeminal neuralgia pain, another was taking pregabalin to treat neuropathic pain, and another was on sildenafil to treat erectile dysfunction.

**Table 1:** Clinical and neuropsychological assessment for each participant group. Values expressed as mean (SD). Statistical comparisons were conducted using independent samples t-tests (df = 56) and the χ2 (df = 1) tests (gender distribution).

	MS patients	Controls	t/ χ^2^	p value
**Demographic variables**				
Gender (men/women)	11/16	12/19	0.169	0.788
Age (years)	34.5 (7.5)	37.5 (8.9)	-1.397	0.168
Years of education	14.4 (2.8)	14.9 (3.0)	-0.627	0.533
**Neurological assessment**				
Disease duration (months)	15.2 (9.4)	-	-	-
Time since last relapse (months)	8.6 (6.4)	-	-	-
EDSS	0.87 (0.91)	-	-	-
MSSS	2.48 (2.22)	-	-	-
**Neuropsychological assessment**				
SRT-Storage	49.6 (11.1)	52.4 (10.1)	-0.990	0.326
SRT-Retrieval	41.4 (12.4)	45.7 (11.5)	-1.348	0.183
SRT-Delayed	10.0 (1.6)	10.0 (1.8)	-0.072	0.943
10/36	24.4 (4.8)	24.7 (4.4)	-0.243	0.809
10/36 Delayed	8.4 (2.1)	8.4 (1.4)	-0.005	0.996
SDMT	62.5 (9.7)	65.7 (8.6)	-1.332	0.188
PASAT 3 seconds	48.6 (7.9)	49.5 (9.0)	-0.380	0.705
PASAT 2 seconds	33.4 (18.5)	37.3 (14.4)	-0.894	0.375
Semantic Fluency Test	21.3 (4.6)	21.3 (3.8)	-0.024	0.981
Phonetic Fluency Test	13.9 (5.6)*	17.0 (3.8)	-2.515	0.015
**Mood and Fatigue assessment**				
BDI	7.0 (7.2)	4.4 (5.2)	1.589	0.118
FSS	2.9 (1.6)	2.3 (1.0)	1.666	0.103

EDSS: Expanded Disability Status Scale; MSSS: Multiple Sclerosis Severity Score; SRT: Selective Reminding Test; 10/36: 10/36 Spatial Recall Test; SDMT: Symbol Digit Modalities Test; PASAT: Paced Auditory Serial Addition Test; Beck: Beck Depression Inventory; FSS: Fatigue Severity Scale

*p<0.05

### Cognitive performance and behavior

As shown in Table 1, no statistically significant differences between groups were found for any of the variables derived from the neuropsychological tests administered, except for phonetic verbal fluency. MS patients scored slightly lower in this test than their matched healthy controls.

No differences were found in scores on the BDI and FSS scales measuring depression and fatigue, respectively.

### ERP analysis of the Stop Task

Table 2 shows the behavioral measures associated with the stop task. No statistically significant differences were found between groups for any of the behavioral measures assessed. The “percentage correct choice responses” refers to stimuli responded to with the correct hand, whereas the “percentage stop signal commission errors” refers to stop signals where the participant failed to withhold a response and produced a button press. “Post-error-slowing” is the time increment observed in correct choice responses when these follow an erroneous trial relative to when they follow a correct trial (correct and error refer in this case to stimulus-response hand assignment).

**Table 2:** Behavioral measures for each participant group in the two neurophysiological tasks. Values expressed as mean (SD). Reaction times (RT) and post-stop-error slowing are expressed in milliseconds. Statistical comparisons were conducted using independent samples t-tests.

	MS patients	Controls	t value (df = 56)	p value
**Stop Task**				
Total responses	585 (41)	586(26)	-0.128	0.898
Percentage of correct choice responses	95.0 (4.6)	93.4 (5.2)	1.270	0.209
^a^RT correct choice responses	474 (53)	460 (55)	1.020	0.312
^b^RT corrected choice errors	300 (145)	279 (174)	0.472	0.638
Percentage stop signal commission errors	48.7 (14.4)	47.0 (16.5)	0.413	0.681
Post-stop-error slowing	44 (41)	34 (41)	0.912	0.366
^a^RT incompatible—compatible trials	29 (13)	31 (16)	-0.594	0.555
% Errors incompatible—compatible trials	3.35 (4.45)	5.96 (5.76)	-1.908	0.062
**Auditory oddball task**				
Percentage non-responded targets	10.47 (9.84)	12.73 (19.87)	-0.538	0.593
Percentage responded novel stimuli	4.23 (3.56)	3.69 (3.09)	0.603	0.545

*p<0.05

^a^RT: reaction time

^b^RT corrected errors: the reaction time taken to correct an erroneous response

**Fig 1:**
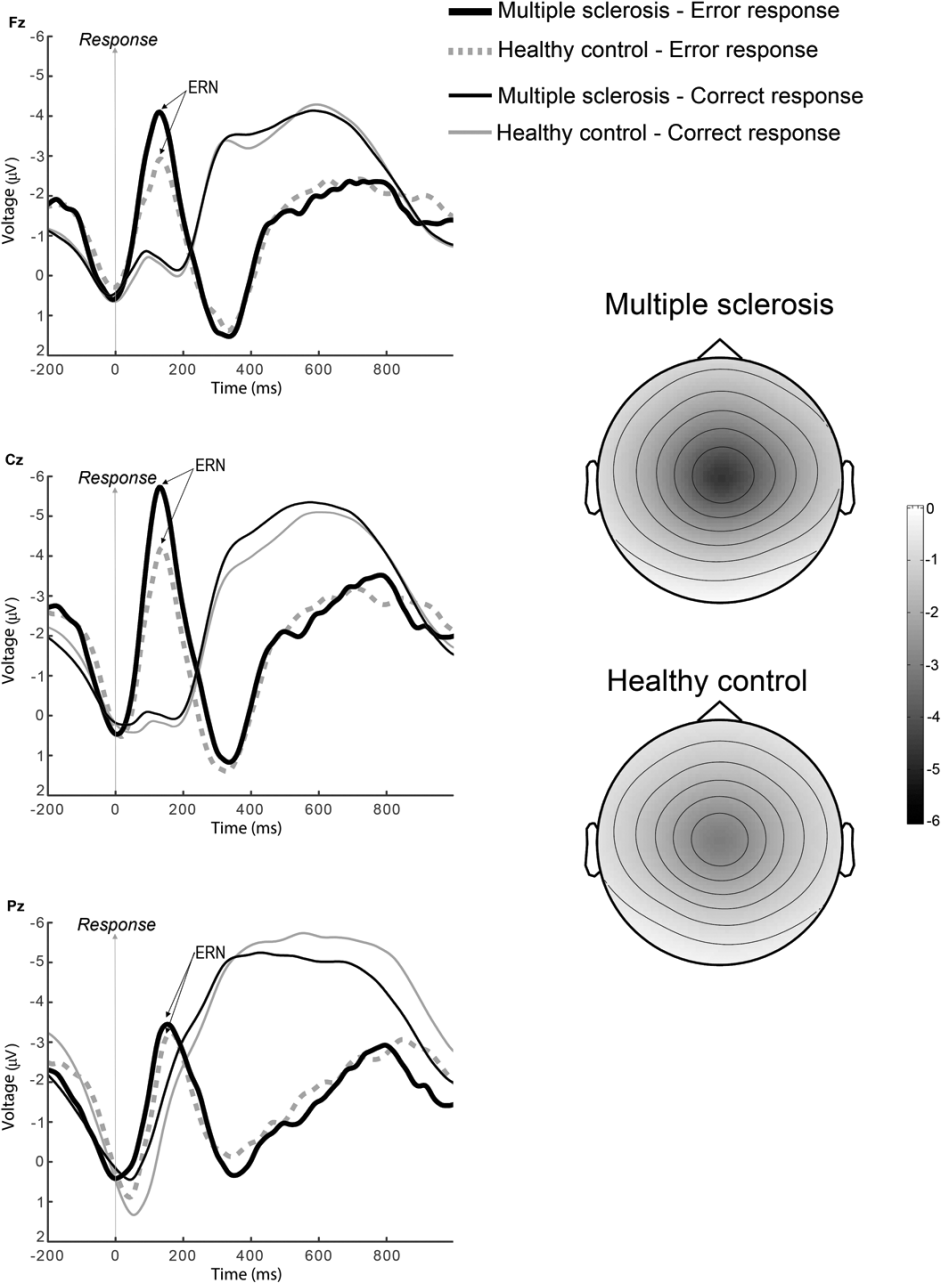
ERPs associated with the stop task. Grand-mean average response-locked ERPs at Fz, Cz and Pz for stop-errors and correctly responded stimuli. The negative-going deflection following a stop error is the ERN. Note the increased amplitude of this wave in the MS patients (solid line) as compared to the healthy controls group (dotted line). The grand-averages have been band-pass filtered (2–8 Hz) for display purposes. The topographical map shows the peak activity of the ERN expressed as the difference wave between error—correct responses. Relative scaling was used. Minimum and maximum values: -5/0 μV.

The preprocessing steps involving eye-blink minimization and artifact correction yielded an epoch rejection of only 3%. The grand averages calculated for each sample in the stop task showed that shortly after the presentation of a stop signal, commission errors were associates with an enhanced negative wave. This wave showed the typical topographical distribution of the ERN with larger values at frontocentral locations. As shown in Fig 1, the grand average of the ERN was larger for the MS group than for the controls. Given that the ERN is a negative component, a larger signal is associated with a more negative value. The topographical map in Fig 1 shows the peak activity of the ERN expressed as the difference wave between error—correct responses.

The two-way ANOVA using the within-subjects Condition factor (correct/error) and the between-subjects Group factor (MS vs. controls) conducted at Fz and Cz did not show an overall effect of Group: Fz [F(1,56) = 3.29, p = 0.075], Cz [F(1,56) = 3.74, p = 0.058]. Nevertheless, it showed significant effects of Condition: Fz [F(1,56) = 30.68, p<0.001], Cz [F(1,56) = 53.64, p<0.001]; and the interaction Group by Condition: Fz [F(1,56) = 4.77, p = 0.033], Cz [F(1,56) = 5.11, p = 0.028]. This interaction indicated a selective effect after the erroneous responses only. Mean voltage values were calculated at the Fz and Cz leads for each participant between 0 and 100 ms following error commission and following correct responses. Mean±SD voltage values following error commission were -2.77±2.70 μV for MS patients and -1.35±2.31 μV for the controls at Fz, and -3.37±2.99 μV for MS patients and -1.61±2.71 μV for the controls at Cz. The statistical comparison between groups (MS vs. controls) showed significant differences at both leads: Fz [t(56) = -2.17, p = 0.034], and Cz [t(56) = -2.35, p = 0.022]. On the contrary, no statistically significant differences were found between groups for correct responses either at Fz [t(56) = -0.508, p = 0.614] or at Cz [t(56) = -0.480, p = 0.633].

The above findings regarding the ERN were confirmed by the alternative approach of measuring peak values in the 0–100 ms time window. Again, MS patients showed larger amplitudes for the ERN than their healthy controls. Peak values (Mean±SD) were -5.47±3.12 μV for MS patients and -3.56±2.83μV for the controls at Fz [t(56) = -2.42, p = 0.019] and -6.91±3.96 μV for MS patients and -4.15±3.56 μV for controls at Cz [t(56) = -2.76, p = 0.008].

To test whether the topographical distribution of the ERN differed between groups, we introduced the mean amplitude from 0 to a 100 ms post error into an ANOVA with electrode (19 levels) and group (MS vs. controls) as factors, and we searched for a significant interaction electrode x group as previously described [25].The ANOVA showed a significant effect of electrode, F(18,1008) = 3.35, p = 0.021, and a significant effect of group, [F(1,56) = 4.391, p = 0.041]. However, it showed a non-significant interaction electrode x group [F(18,1008) = 1.034, p = 0.378]. Thus, the ERN topographies were not significantly different between groups.

### ERP analysis of the Auditory oddball task

Table 2 shows the behavioral measures associated with the auditory oddball task. No statistically significant differences were found between groups for any of the behavioral measures assessed.

**Fig 2:**
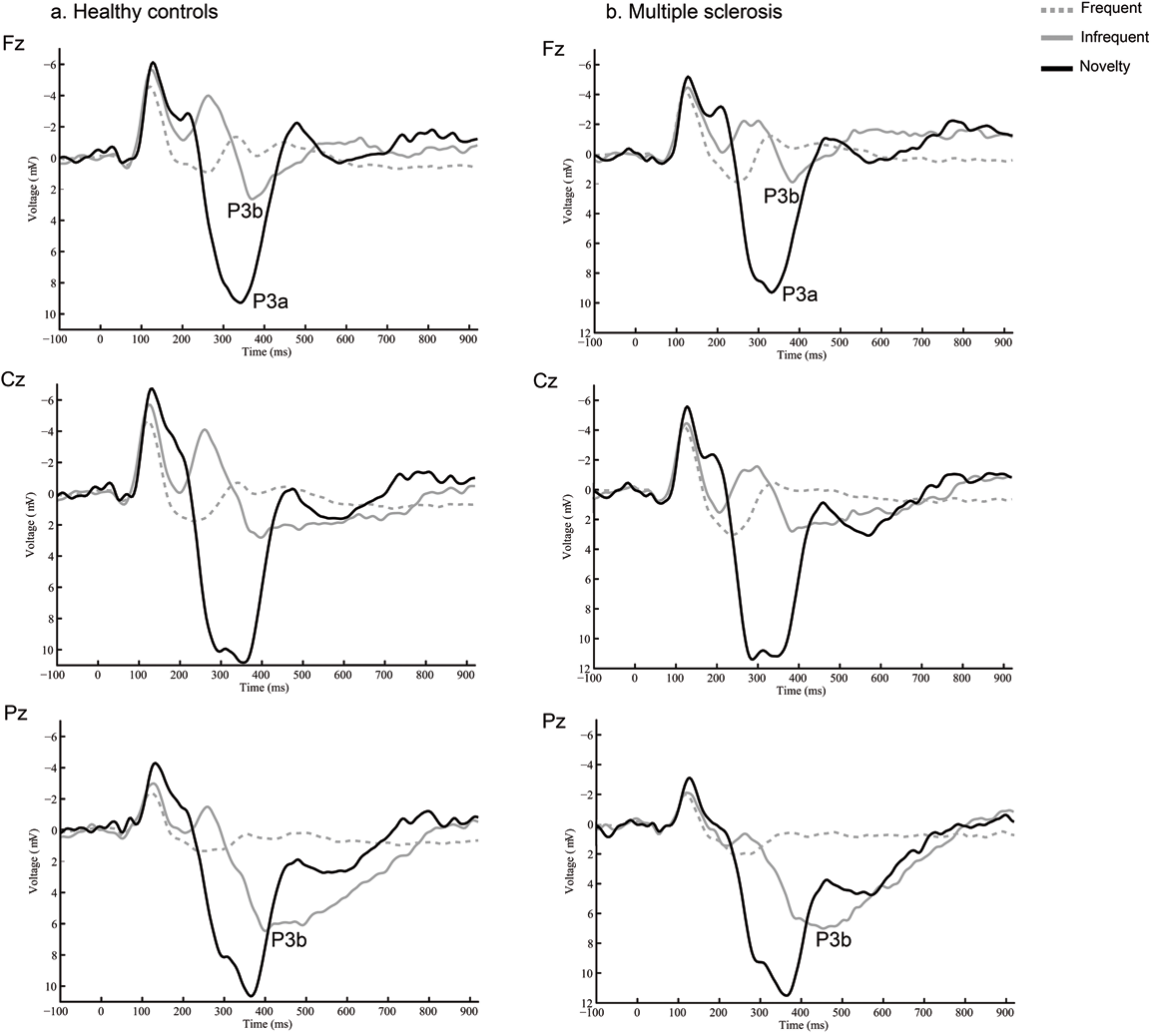
ERPs associated with auditory the oddball task. Grand-average stimulus-locked ERPs at Fz, Cz and Pz following the presentation of standard tones (solid black lines), target tones (solid grey lines), and novel tones (dotted grey lines). Amplitude of the P3b component (target tones) was maximum at Pz, whereas amplitude of the P3a component (novel tones) was maximum at Cz. No differences in amplitudes or latencies were found between MS patients and controls.

The preprocessing steps involving eye-blink minimization and artifact correction yielded an epoch rejection of only 2%. Fig 2 illustrates ERPs associated with the standard, target, and novel stimuli in the auditory oddball task. A centro-parietal P3b component is present in the target waveforms, whereas a more frontally distributed P3a is seen for the novel stimuli. Visual inspection of the grand averages did not reveal any differences between MS patients and controls. This was confirmed by the statistical analyses of the latency and magnitude of the ERP components.

The two-way ANOVA with the within-subjects Condition factor (standard/target/novel) and the between-subjects Group factor (MS vs. controls) conducted at Fz, Cz and Pz for peak latency showed significant effects of Condition: Fz [F(2,112) = 172, p<0.001], Cz [F(2,112) = 187, p<0.001], Pz [F(2,112) = 196, p<0.001]. No effect of Group was observed: Fz [F(1,56) = 0.696, p = 0.408], Cz [F(1,56) = 0.028, p = 0.869], Pz [F(1,56) = 0.004, p = 0.947]; nor their interaction: Fz [F(2,112) = 1.120, p = 0.319], Cz [F(2,112) = 0.856, p = 0.405], Pz [F(2,112) = 0.535, p = 0.568]. Mean±SD values for P3a latency at Cz were 331±40 ms for patients and 324±49 ms for controls [t(56) = 0.57, p = 0.567]. Regarding the P3b, mean±SD latency values at Pz were 464±63 ms for patients and 454±69 ms for controls [t(56) = 0.57, p = 0.571].

The two-way ANOVA with the within-subjects Condition factor (standard/target/novel) and the between-subjects Group factor (MS vs. controls) conducted at Fz, Cz and Pz for peak amplitude showed a significant effect of Condition: Fz [F(2,112) = 165, p<0.001], Cz [F(2,112) = 180, p<0.001], Pz [F(2,112) = 210, p<0.001]. No effect of Group was found: Fz [F(1,56) = 0.200, p = 0.656], Cz [F(1,56) = 0.008, p = 0.929], Pz [F(1,56) = 0.673, p = 0.415]; nor their interaction: Fz [F(2,112) = 1.61, p = 0.209], Cz [F(2,112) = 1.030, p = 0.355], Pz [F(2,112) = 0.048, p = 0.947]. Mean±SD values for P3a amplitude at Cz were 13.88 ± 6.40 μV for patients and 13.77 ± 4.75 μV for controls [t(56) = 0.07, p = 0.946]. Mean±SD values for P3b amplitude at Pz were 8.95±4.52 μV for patients and 8.39±4.00 μV for controls [t(56) = 0.50, p = 0.620].

### Correlations

**Fig 3:**
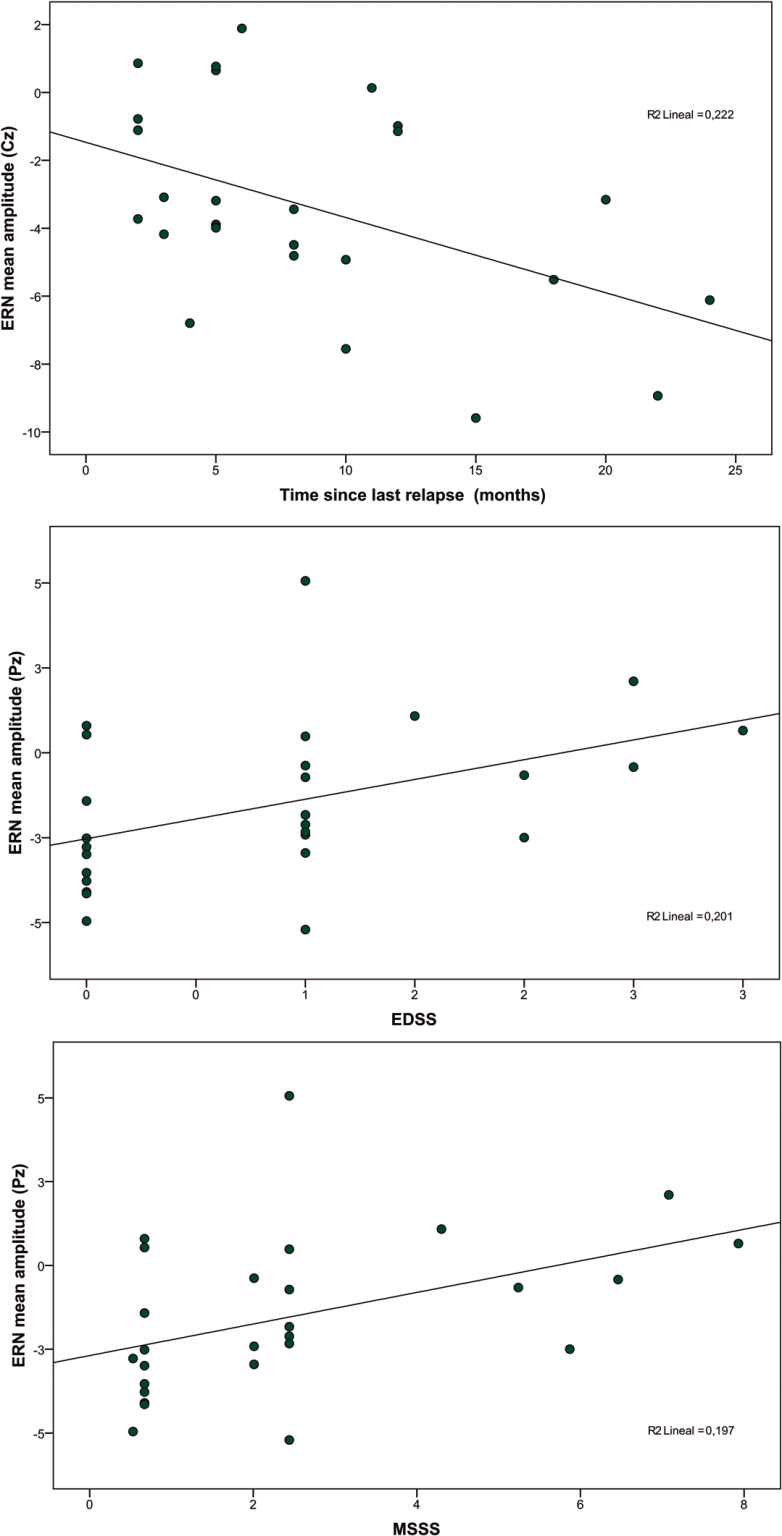
Upper panel: Correlation between amplitude of the ERN at the Cz electrode and time since last relapse. Middle panel: Correlation between amplitude of the ERN at the Pz electrode and score on the Expanded Disability Status Scale (EDSS). Lower panel: Correlation between amplitude of the ERN at the Pz electrode and score on the Multiple Sclerosis Severity Score (MSSS). Data from the 27 MS patients.

Statistically significant correlations were seen between ERN amplitude values and scores on several clinical parameters. We found a significant negative correlation between time since last relapse and amplitude of the negativity at Cz [r = -0.472, r^2^ = 0.223, p = 0.013] and Pz [r = -0.383, r^2^ = 0.147, p = 0.048]. That is, the larger the ERN (the more negative its value), the more time had passed since the last relapse. Additionally, we found a significant positive correlation between the EDSS score and amplitude of the ERN at Cz [r = 0.400, r^2^ = 0.160, p = 0.039] and Pz [r = 0.449, r^2^ = 0.202 p = 0.019]; and between the MSSS and amplitude of the ERN at Cz [r = 0.388, r^2^ = 0.151, p = 0.045] and Pz [r = 0.443, r^2^ = 0.197 p = 0.021] That is, the smaller the ERN (the less negative its value), the higher the impairment. Scatterplots for these correlations are shown in Fig 3.

## Discussion

Using a behavioral stop-task associated with ERP recordings, our results showed group differences in amplitude of the negative-going deflection (ERN) found in trials where subjects failed to inhibit their responses following a stop signal. Immediately after the commission error, the ERN was observed in all participants at frontocentral leads replicating studies by other researchers [26]. This ERP component is considered to be a neurophysiological index of error detection [12] and conflict monitoring [27], two important aspects of executive function [28].

Early stage MS patients showed higher (more negative) ERN amplitudes than controls in the absence of any behavioral differences. In other words, analogous performance was associated with greater neurophysiological activation in the patients. The ERN proved to be a sensitive neurophysiological marker, detecting group differences in a sample carefully selected to rule out differences in education, mood or fatigue. Interestingly, the amplitude of the signal was not smaller in the patient group, but significantly larger. This patient population would thus appear to need larger neural recruitment to maintain performance levels analogous to those of the healthy population.

MS is a progressive disease associated with gradual cognitive deterioration. This process leads to impairment in many spheres of cognitive functioning such as attention, language and executive function [29]. Interestingly, the present neurophysiological findings were seen in patients at an early stage of the disease, when most neuropsychological tests did not yet find any impairment.

The compromise of cognitive function in MS has received increasing attention in recent years due to its high prevalence. From a neuropsychological approach, a comprehensive cognitive battery is needed to assess these deficits. In a study by Glanz and colleague [30], 49% of patients with clinically isolated syndrome or newly diagnosed MS showed impairment in at least one cognitive measure, compared to 30% of healthy controls. Cognitive deficits were observed mainly in sustained attention, processing speed and verbal memory. Deloire et al. found that 59.7% of recently diagnosed MS patients scored below the fifth percentile of the performance distribution of the normal population in two or more tests [31]. In the same study, when patients were asked about their perceived impairment, only 10% of the subjects did not refer any complaint. However, many respondents considered their cognitive problems to be moderate and described them as rarely noticed by others. In contrast with these findings, the results of the present study only showed differences between controls and patients in the phonetic fluency test. Lack of differences in other tests may be due to patients’ cognitive preservation [32], or to the presence of compensatory mechanisms [9].

Other researchers have used ERPs in the past to assess the compromise of cognitive processing in MS. A study involving different illness subtypes reported that around 56% of patients had pathologically increased P3b latencies. These increases were mainly observed in secondary progressive multiple sclerosis [33]. Aminoff and colleagues described electrophysiological changes in MS patients that correlated with cognitive status, and proposed the use of ERPs for the assessment of cognitive function [34]. In another study involving a large sample of patients and a choice reaction time task, researchers unexpectedly found P300 amplitude was high in frontal leads compared to controls. Whereas a positive correlation was found between amplitude of the P300 and performance in the patient subgroup, none was found in the control group [10]. The authors interpreted results in the patient population as showing a compensatory mechanism relying on increased frontal activation.

Additional support for a compensatory mechanism has been provided by neuroimaging studies [8,9,35]. One fMRI study assessed brain activation in MS patients while they performed an attentional task. A cognitively preserved subgroup showed behavioral performance that was analogous to that of controls, but the intensity and extension of the fMRI BOLD response was greater. It involved additional activation clusters in numerous brain regions: the inferior and superior frontal gyri, the dorsolateral prefrontal cortex and the anterior cingulate gyrus [9]. All these areas play prominent roles in executive function in general and performance monitoring in particular [11]. In another study, the authors administered the Paced Auditory Serial Addition Test to MS patients with mild cognitive impairment. Those patients with preserved behavioral performance (i.e., analogous to that of the control group) also showed hyperactivity and more widespread recruitment of brain areas, especially in frontal regions. This effect was less intense in the patient subsample that showed poor performance (i.e., worse than that of the controls) Furthermore, increased frontal activation was positively correlated with lesion burden, as measured by T2 MRI in the overall patient group [35].

Interestingly, in the present study no differences were observed between groups in the relatively simple auditory oddball task. Latency and amplitudes of the P3a and P3b components of the ERP were not altered in our sample. No modifications were thus observed in stimulus categorization (P3b) or in the frontal attentional network (P3a). These processes are also thought to rely on the ACC but in contrast to performance monitoring they are less cognitively demanding [36,37].

On the contrary, enhanced activation was observed in association with error detection. This ability is a crucial aspect of performance monitoring. It plays a major role when addressing environmental demands and guides goal-directed behavior. As proposed by Penner and colleagues, increased neural recruitment would precede cognitive decline. The latter would become manifest when compensatory mechanisms are exhausted [9]. A dramatic deterioration would be observed later on, usually in the course of the subsequent five years of the disease [29]. At later stages, the more automatic and less demanding orienting response would be affected, and the P3a and P3b would show increased latencies and smaller amplitudes [10].

The above interpretation is supported by the results of the correlation analysis. The more negative or larger the ERN, that is, the greater the neural recruitment, the longer the patients stayed relapse-free. On the contrary, those patients showing smaller (less negative) ERN values had greater EDSS and MSSS scores, indicating a worse clinical status. In line with these findings, a neurophysiological study measuring the P300 found increased ERP in patients relative to controls [10]. Furthermore, cognitive performance was positively correlated with P300 amplitude, and patients displaying low amplitudes were more cognitively impaired. No such correlation between P300 amplitude and performance was observed in the control group.

The present study has several limitations. First, an enhanced ERN can be detected in other pathologies, not only in MS. Second, some patients were receiving pharmacological treatment while others were not. Finally, it cannot be entirely ruled out that the diverse nature of MS manifestations may affect the ERN.

To sum up, in the present study we found increased amplitude of the ERN, a neurophysiological correlate of performance monitoring, in a group of early-stage MS patients. This enhanced signal was observed in the absence of behavioral and cognitive differences between groups. These findings can be interpreted in terms of greater neuronal recruitment in the patient group and indicate early compensatory mechanisms in MS. Our results highlight the sensitivity of ERN to detect early differences in cognitive processes in this sample.

## Supporting Information

S1 Dataset Data base with neurophysiological and neuropsychological data. (SAV)
